# In Vitro Nephrotoxicity Studies of Established and Experimental Platinum-Based Compounds

**DOI:** 10.3390/biomedicines9081033

**Published:** 2021-08-18

**Authors:** Sarah Schoch, Vasily Sen, Walburgis Brenner, Andrea Hartwig, Beate Köberle

**Affiliations:** 1 Department of Laboratory Medicine, Lund University, Scheelevägen 2, 223 81 Lund, Sweden; sarah.schoch@med.lu.se; 2 Institute of Problems of Chemical Physics, Russian Academy of Sciences, Chernogolovka, 142432 Moscow, Russia; senvd@icp.ac.ru; 3 Clinic for Obstetrics and Women’s Health, Department of Urology and Pediatric Urology, University Medical Center Mainz, Langenbeckstreet 1, 55131 Mainz, Germany; walburgis.brenner@unimedizin-mainz.de; 4 Karlsruhe Institute of Technology, Department of Food Chemistry and Toxicology, Adenauerring 20, 76131 Karlsruhe, Germany; andrea.hartwig@kit.edu

**Keywords:** cisplatin, platinum drugs, DNA damage response, nephrotoxicity, chemotherapeutic drugs, gene expression profiling

## Abstract

Cisplatin is one of the most commonly used drugs for the treatment of various solid cancers. However, its efficacy is restricted by severe side effects, especially dose-limiting nephrotoxicity. New platinum-based compounds are designed to overcome this limitation. Previous investigations showed that the platinum(IV)–nitroxyl complex PN149 is highly cytotoxic in various tumor cell lines. In the present study, investigations with PN149 were extended to normal human kidney tubule epithelia. Coincident with higher intracellular platinum accumulation, the cytotoxicity of PN149 in the proximal tubule epithelial cell line ciPTEC was more pronounced compared to the established platinum chemotherapeutics cisplatin, carboplatin and oxaliplatin. Quantitative gene expression profiling revealed the induction of ROS-inducible and anti-oxidative genes, suggesting an oxidative stress response by PN149. However, in contrast to cisplatin, no pro-inflammatory response was observed. Genes coding for distinct DNA damage response factors and genes related to apoptosis were up-regulated, indicating the activation of the DNA damage response system and induction of the apoptotic cascade by PN149. Altogether, a comparable transcriptional response was observed for PN149 and the platinum chemotherapeutics. However, the lack of inflammatory activity, which is a possible cause contributing to toxicity in human renal proximal tubule epithelia, might indicate the reduced nephrotoxic potential of PN149.

## 1. Introduction

**Figure 1:**
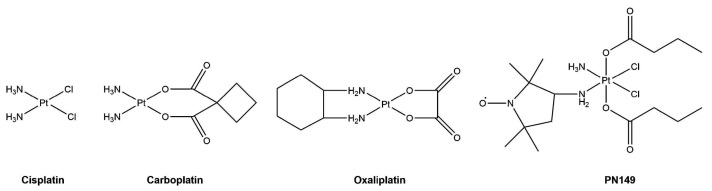
Structure of the platinum compounds cisplatin, carboplatin, oxaliplatin and PN149.

Cisplatin (Figure 1) is one of the most commonly used drugs in chemotherapy. It is applied against a variety of cancers such as bladder, lung and ovarian cancer and carcinomas of the head and neck [1]. Cisplatin is particularly effective in combination therapy against testicular germ cell tumors, where cure rates of over 80% are achieved [2]. However, its clinical application is restricted by tumor cell resistance, either intrinsic to the tumor or acquired during cycles of cisplatin therapy, and severe side effects, especially dose-limiting nephrotoxicity [3]. One-third of patients receiving cisplatin therapy suffer severe renal toxicity, which is commonly manifested as acute kidney injury (AKI) [4].

The mechanisms of the toxic activity of cisplatin have been thoroughly investigated in various cancer cell lines [5,6,7,8]. Cisplatin enters the cells by both passive diffusion and active transport mechanisms, mediated by cellular membrane transporters [9,10]. Upon entering the cells, cisplatin becomes activated by the replacement of the two chloride leaving groups by water and subsequently binds covalently to DNA, resulting in various DNA platinum lesions. DNA platination activates a number of signal transduction pathways that control cell cycle arrest/DNA repair or apoptosis. Depending on the extent of DNA damage, cell cycle arrest will be induced to ensure time for the repair of platinum damage, or, in case of excess damage, platinum lesions will trigger apoptosis, which is thought to be crucial for cisplatin toxicity and hence therapeutic efficacy in cancer cells, but also for the toxic side effects in normal cells [8,11]. The mechanisms underlying cisplatin-induced nephrotoxicity are not fully understood, but besides cisplatin-induced DNA damage response (DDR) and apoptosis, the generation of reactive oxygen species (ROS) and inflammatory processes are possible causes contributing to cisplatin toxicity in kidney cells [4,12,13,14,15,16]. Furthermore, the binding of cisplatin to the intracellular thiol-containing molecule glutathione might result in the production of a nephrotoxic intermediate that could also contribute to the dose-restricting toxic side effect [17,18,19].

One approach to address the limitations connected with cisplatin treatment is the design of new, better tolerated cisplatin analogues that hold lower toxicity but retain antitumor activity and might even display activity in cisplatin-resistant cancers [11]. Modifications of the structure of cisplatin led to the development of numerous platinum analogues, which have been tested in cell cultures and pre-clinical cancer models [20]. So far, carboplatin and oxaliplatin (Figure 1) have achieved worldwide approval for chemotherapeutic treatment. Carboplatin is less toxic than cisplatin and shows fewer side effects, with severe myelosuppression being dose-limiting for therapeutic treatment. However, carboplatin shows no activity in cisplatin-resistant tumor cells in vitro and is used against essentially the same tumor types as cisplatin in the clinic. Oxaliplatin, on the other hand, has a different pattern of sensitivity compared to cisplatin and carboplatin. In numerous clinical trials, oxaliplatin showed activity in cisplatin-resistant colorectal cancer patients, resulting in clinical approval for the treatment of advanced colorectal cancer, which is insensitive towards cisplatin treatment [11,21,22]. With respect to side effects, oxaliplatin treatment leads to severe neurotoxicity as a dose-limiting side effect [3].

To broaden the spectrum of clinical application to additional tumor types, the search for cisplatin analogues continues, with new platinum compounds being designed and tested in pre-clinical and clinical investigations [20,23]. Of particular interest are platinum(IV) complexes, as they might possess sufficient oral bioavailability for oral platinum chemotherapy [24]. Platinum(IV)–nitroxyl complexes (PNCs) are hybrid compounds combining platinum with biologically active nitroxyl pharmacophores (Figure 1) [25,26,27]. Due to the two additional axial ligands, PNCs are highly lipophilic, which allows easy uptake by cells. We found that PNCs were cytotoxic in tumor cell lines of different origin [28,29]. Furthermore, we observed that PNCs were still active in cisplatin-resistant bladder and testis tumor cells, indicating that PNCs were able to circumvent the cisplatin resistance phenotype in vitro [28]. This observation could be highly relevant for potential clinical use in tumors that are unresponsive to cisplatin treatment. Generally, PNCs share a comparable mode of action to that of cisplatin in tumor cells, as demonstrated for the PNC PN149 [*e*-ammine-*d*-(3-amino-2,2,5,5-tetramethylpyrrolidine-1-oxyl)-*a,f*-bis(butanoato)-*b,c*-dichloro-platinum(IV)] in the RT112 bladder cancer cell line and A498 kidney cancer cell line. However, an inflammatory response was restricted to RT112 cells, but was not observed in A498 kidney cancer cells [29]. As inflammatory processes have been implicated with cisplatin-induced nephrotoxicity, the lack of an inflammatory response following PN149 treatment in kidney cancer cells suggested a less nephrotoxic potential of PN149. To elucidate the mode of action of PN149 in normal human kidney cells in comparison to the clinically approved platinum chemotherapeutics cisplatin, carboplatin and oxaliplatin, gene expression analysis was applied [30]. Selected genes related to transcription factors, (oxidative) stress response, DNA damage response/repair, cell cycle control and apoptosis were chosen for the analysis. As a model system for the investigations in kidney cells, the renal cell line ciPTEC, which is derived from human proximal tubule epithelial cells, was used [31].

## 2. Materials and Methods

### 2.1. Cell Culture

The renal cell line ciPTEC (conditionally immortalized proximal tubule epithelial cells) [31], which was purchased from Dr. Martijn Wilmer, was used in the current study. ciPTECs were cultured in DMEM-HAM’s F12 phenol red-free medium (Gibco, Thermo Fisher Scientific, Dreieich, Germany) supplemented with 10% fetal calf serum (FCS) (Invitrogen, Darmstadt, Germany), 5 µg/mL insulin, 5 µg/mL transferrin, 5 ng/mL selenium, 36 ng/mL hydrocortisone, 10 ng/mL EGF (epidermal growth factor) and 40 pg/mL tri-iodothyronine (Sigma-Aldrich, Steinheim, Germany). For maintaining the proliferation status, cells were cultured at 33 °C in a humidified atmosphere of 5% CO_2_ in medium supplemented with 1% penicillin/streptomycin (P/S) (Sigma-Aldrich, Steinheim, Germany). For the experiments, ciPTECs were seeded at a density of 55,000 cells/cm^2^ in cell culture flasks and were cultivated for 24 h at 33 °C. After 24 h, cells were transferred to 37 °C for maturation, while the medium was refreshed with medium containing no P/S. Cells were kept at 37 °C for 7 days while the medium was replaced every 2–3 days. For comparative analysis with the data obtained with the permanent cell line ciPTEC, studies were performed with primary patient-derived renal normal tissue cells (NT), which were obtained from patients at the University Medical Center Mainz. Cells were isolated from primary renal cancer samples of patients who underwent nephrectomy at the Department of Urology, University Medical Center Mainz. The study was performed in agreement with the Declaration of Helsinki and approved by local ethics committee (No. 837.005.09, Landesärztekammer Rheinland-Pfalz, Mainz, Germany). Each patient provided informed consent. NT cells were cultivated in RPMI-1640 medium (Sigma-Aldrich, Steinheim, Germany) containing 10% FCS and 5% P/S at 37 °C in a humidified atmosphere of 5% CO_2._

### 2.2. Platinum-Based Compounds

The three clinically approved platinum-based compounds cisplatin, carboplatin and oxaliplatin and the experimental drug PN149 were used. An infusion solution of cisplatin with a concentration of 1 g/L was kindly provided by the Clinical Centre Karlsruhe. Infusion solutions of carboplatin and oxaliplatin were purchased from Accord Healthcare Limited (Middlesex, UK) with a respective concentration of 10 mg/mL and 5 mg/mL. PN149 was synthesized at the Institute of Problems of Chemical Physics of the Russian Academy of Sciences. Incubation times with the platinum compounds were based on the excretion time of cisplatin in patients. Cisplatin shows a 3 phase kinetic excretion with a first half-life time (t1/2α) of 14–49 min followed by a second half-life period (t1/2β) of 0.7–4.6 h and a third half-life period (t1/2γ) of 24–127 h [32,33]. To be in line with the clinical conditions, an incubation time of 24 h was chosen for the experiments, with the exception of intracellular platinum accumulation studies, which were performed for 2 h. Concentrations of the compounds were chosen based on the blood plasma levels measured in patients after drug treatment. The concentration of cisplatin ranged from 10–50 µM. Due to its slower reaction kinetics compared to cisplatin, the concentration of carboplatin ranged from 100 to 500 µM, whereas oxaliplatin is clinically used in a similar range to cisplatin [32,34].

### 2.3. Cytotoxicity Studies

Toxicity was determined by relative cell count (RCC). ciPTECs were seeded in duplicates in T25 flasks and cultivated as stated before. After maturation, cells were incubated for 24 h with cisplatin (10, 20, 50 µM), PN149 (10, 20, 50 µM), carboplatin (100, 200, 500 µM) or oxaliplatin (20, 50, 100 and 150 µM) followed by a post-cultivation period of 48 h. Cell count was then determined using a CASY^®^ cell counter (CASY^®^ TTC Cell Counter & Analyzer System). The cell counts of the treated samples were normalized to the untreated control to obtain the RCC expressed as percentage. RCC was determined in three independent experiments.

### 2.4. Intracellular Platinum Accumulation

Atomic absorption spectroscopy (AAS) was used to determine the intracellular accumulation after treatment with the platinum compounds. ciPTECs were seeded in T75 flasks and cultivated as stated before. Matured cells were treated for 2 h with 50 µM of cisplatin, oxaliplatin or PN149 or 300 µM carboplatin. Cells were then harvested via centrifugation and total cell count was determined. Afterwards, cell pellets underwent an acidic digestion using a solution of 30% H_2_O_2_ and 65% HNO_3_ (1:1 (*v*:*v*)) (Roth, Karlsruhe, Germany) and evaporation. The residue was dissolved in 0.2% HNO_3_ and platinum amount was analyzed at a wavelength of 26,594 nm in a graphite furnace using a PinAAcle 900 T (Perkin Elmer, Waltham, MA, USA). Atomization was performed using a furnace temperature program consisting of two drying steps of 120 °C for 30 s and 140 °C for 45 s followed by a pyrolysis step of 1300 °C for 20 s, an atomization step of 2400 °C for 5 s as well as a heating step for clean out of 2500 °C for 5 s. Intracellular accumulation was determined in three independent experiments and was calculated as ng Pt/10^6^ cells.

### 2.5. Gene Expression Profiling by High-Throughput RT-qPCR

Gene expression profiles were generated using a high-throughput RT-qPCR method previously established in the institute [30]. ciPTECs were cultivated in T25 flasks as stated before. Matured cells were incubated for 24 h with cisplatin or PN149 (10, 20, 50 µM), carboplatin (100, 200, 500 µM) or oxaliplatin (20, 50, 100 and 150 µM) and subsequently harvested via centrifugation. RNA isolation and generation of gene expression profiles using a Fluidigm dynamic array on a BioMark™ (Rheinau, Germany) system were performed as previously described [29]. For comparative analysis of ciPTEC with primary kidney cells, 5 × 10^5^ NT cells were seeded in 10 cm dishes and cultivated for 24 h. Afterwards, cells were incubated with 20 µM cisplatin for 24 h followed by harvesting the cells via centrifugation and generation of gene expression profiles. Gene expression profiles in ciPTEC were generated in three independent experiments. Due to the limited amount of tissue material, gene expression analysis in primary kidney cells was performed in two independent experiments.

### 2.6. Spectrophotometric Measurement of Anti-Oxidant Capacity of Platinum Compounds

The influence of PN149 on the rates of the reaction of fluorescein with free radicals generated by the azo initiator 2,2′-azobis(2-amidinopropane) dihydrochloride (AAPH) was studied according to [35] in phosphate buffer (PB, 50 mM, pH 7.4) by measuring the decay of the probe at 494 nm with absorption spectrophotometer Specord UV–Vis equipped with a thermostated cell maintained at 37 °C. The reaction was started by the addition of AAPH to a preheated solution of fluorescein or fluorescein plus cisplatin or PN149 in PB. The lag phase of the reaction means no changes in the optical density of the reaction mixture. AAPH and fluorescein were purchased from Sigma-Aldrich, JM216 [*e*-ammine-*d*-(cyclohexylamine)-*a,f*-bis(acetato)-*b,c*-dichloroplatinum(IV)] was obtained as described in [36].

## 3. Results and Discussion

### 3.1. Experimental Models for In Vitro Nephrotoxicity Studies 

With respect to nephrotoxicity investigations, choosing the right experimental model is a challenging task. Potential new drugs often fail in clinical phase 1 and 2 safety assessments due to nephrotoxicity that was not detected in previous preclinical studies. Many in vitro as well as in vivo models proved to be unsuitable or unreliable to detect whether a drug has the potential to be nephrotoxic to humans [37]. Membrane transporters, which play a critical role in the impact of drugs and chemical compounds on kidney cells, are often differently expressed in animal kidneys compared to the human kidney [38]. Therefore, animal models should be used with caution for nephrotoxicity studies. Patient-derived primary cells would be an ideal choice as they reflect the in vivo situation in the human body. However, their availability is limited, inter-donor variability is high and primary cells de-differentiate during passaging, resulting in reduced expression levels of some membrane transporters and enzymes [39]. The use of permanent cell lines established from human kidney cells would allow us to circumvent these limitations. One cell line, which has been frequently used for nephrotoxicity studies over the years, is the human embryonic kidney 293 (HEK293) cell line. However, it turned out that HEK293 might not be of renal epithelial origin but is related to neuronal cells [40]. Another cell line frequently used for nephrotoxicity studies is HK-2, an immortalized cell line of proximal tubule origin. However, HK-2 cells have some drawbacks, as they retained only limited proximal tubule functions, with only low expression levels of transporters of the solute carrier (SLC) family, which are responsible for cisplatin uptake into proximal tubule cells [41,42,43]. More recent kidney cell culture models, which retained their proximal tubule characteristics and functionalities in vitro, are the cell lines RPTEC/TERT1 and ciPTEC [31,44]. RPTEC/TERT1 is derived from proximal tubule cells that were infected with a vector containing hTERT cDNA [44]. The ciPTEC cell line was established by Wilmer and colleagues by infecting primary renal cells with vectors containing the temperature-sensitive mutant of SV40 large T antigen (SV40T) and the catalytic subunit of human telomerase (hTERT) [31]. In this conditionally immortalized proximal tubule cell line, proliferation is maintained at the permissive low temperature of 33 °C, while cells transferred to 37 °C grow into tight confluent monolayers with functional membrane transporters involved in renal reabsorption and excretion. ciPTECs were therefore used as a model for human renal proximal tubular epithelial cells to study the activity of PN149 compared to the established chemotherapeutic platinum drugs in human kidney cells.

### 3.2. Cytotoxic Potential of Platinum Compounds in ciPTEC 

**Figure 2:**
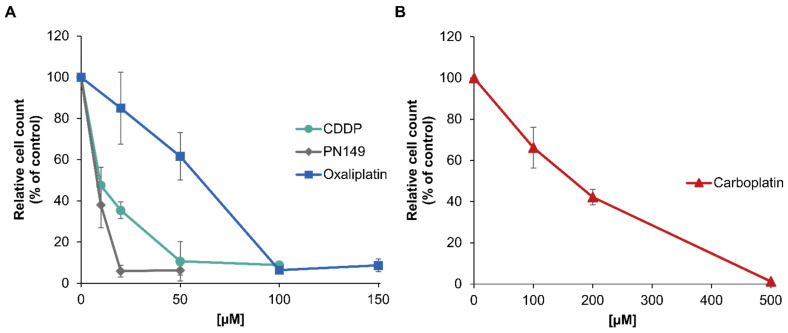
Cytotoxicity of PN149, cisplatin, oxaliplatin or carboplatin in the renal cell line ciPTEC. ciPTECs were incubated with (**A**) PN149, cisplatin or oxaliplatin or (**B**) carboplatin at the indicated concentrations for 24 h, and the cell count was determined after 48 h post-cultivation. Relative cell count (RCC) was obtained by normalization to the untreated control. Shown are the mean values of three independent experiments ± standard deviation.

The cytotoxicity of PN149 in comparison to cisplatin, carboplatin and oxaliplatin was determined by assessing cell viability through cell number. Differentiated ciPTECs were incubated for 24 h with different concentrations of the respective platinum complexes and cell count was measured after a post-cultivation period of 48 h. PN149 led to a strong decrease in cell number, resulting in a reduction to less than 5% compared to the control (Figure 2A). The pronounced cytotoxicity of PN149 is reflected by an IC_50_ value of 6 µM (Table 1). A decrease in cell number was also observed for the other platinum compounds, with cisplatin being slightly less cytotoxic than PN149, with a pronounced reduction in cell number to less than 10% at 50 µM and an IC_50_ value of 13 µM. In contrast to these findings in normal kidney cells, PN149 and cisplatin show comparable toxicity in cancerous cell lines [28,29].

Moderate cytotoxicity was observed for oxaliplatin (IC_50_ = 51 µM), while carboplatin was only weakly cytotoxic and had to be applied at a concentration 10 times higher to evoke a cytotoxic response (IC_50_ = 175) (Figure 2B, Table 1). Therefore, even though all platinum compounds were cytotoxic in proximal tubule cells, the extent of toxicity differed substantially. A cytotoxic impact on ciPTECs was also observed for cisplatin by Nieskens and colleagues [45]. Using the MTT assay for toxicity studies, they calculated a TC_50_ (≡ IC_50_) of 34 µM cisplatin in ciPTECs treated with cisplatin for 24 h. Secker and colleagues, on the other hand, did not observe any toxicity after 24 h incubation with cisplatin (10–100 µM) in the 2D cell culture model of RPTEC/TERT1 tubule cells using the LDH assay [46]. However, by extending the post-cultivation time up to 11 days, a cytotoxic impact was observed for the highest cisplatin concentration used (100 µM) [46]. The discrepancy between the observations in ciPTEC versus RPTEC/TERT1 might be at least in part due to the different assays used to investigate cisplatin toxicity. However, regardless of the discrepancy in observations, our data revealed that oxaliplatin and carboplatin were also toxic in an in vitro model of proximal tubule cells. These findings do not reflect the clinical situation, as nephrotoxicity is not observed as an unwanted side effect for chemotherapeutic treatment with carboplatin or oxaliplatin. Unfortunately, no comparable studies about the effect of carboplatin and oxaliplatin in ciPTEC or RPTEC/TERT1 human proximal tubule cells have been performed to date. Few studies investigated the impact of carboplatin and oxaliplatin in comparison to cisplatin in renal cell lines. In the MDCK dog kidney cell line, a strong toxic effect was observed for the three clinically approved platinum compounds [47]. Similarly, high toxicity following treatment with the three compounds was shown in the rat renal proximal tubular epithelial cell line NRK-52E [48]. In vivo studies, however, revealed renal damage induced by cisplatin, but not by carboplatin and oxaliplatin [49], demonstrating again the challenges of nephrotoxicity studies. In the present in vitro studies using a model system for human proximal tubule cells, the most pronounced cytotoxicity was observed for the experimental compound PN149, which one might consider as an indication of possible nephrotoxicity. However, as the cell culture observations of carboplatin and oxaliplatin do not reflect the clinical situation, one cannot rule out a different in vivo response of PN149 in the human kidney. Altogether, the observations reflect the problem of performing nephrotoxicity studies using in vitro models. Nevertheless, one could argue that investigating only acute toxicity is not sufficient for analyzing the nephrotoxic potential of a drug. Furthermore, elucidating the mode of action of different platinum compounds in normal kidney cells might help to design new and improved platinum drugs. Therefore, further studies regarding intracellular platinum accumulation and detailed gene profiling were carried out.

### 3.3. Intracellular Platinum Accumulation

The main targets of the toxic effects of cisplatin in the kidneys are epithelial cells located in the S3 segment of the proximal tubule. The kidney is the major excretory organ of cisplatin, hence a five times higher accumulation of the drug has been observed in epithelial cells of the proximal tubule compared to blood plasma concentrations [50,51]. Furthermore, the activity of specific transporters in the membranes of epithelial cells, especially of the solute carrier (SLC) family, is responsible for its high uptake in proximal tubule epithelial cells (PTECs) [42,43]. Carboplatin and oxaliplatin, on the other hand, have little or no nephrotoxic potential, which might be due to the observation that carboplatin and oxaliplatin show little accumulation in nephron cells [52]. To compare the intracellular accumulation of cisplatin, carboplatin and oxaliplatin in comparison to PN149 in kidney tubule cells, ciPTECs were incubated for 2 h with 50 µM of either PN149, cisplatin or oxaliplatin or 300 µM carboplatin, and the platinum amount was measured directly after treatment by AAS. The chemotherapeutics cisplatin, oxaliplatin and carboplatin showed comparable amounts of platinum accumulation, while for PN149 are about 20 times higher platinum accumulation was observed (Table 1). The stronger cytotoxicity of PN149 compared to cisplatin, carboplatin and oxaliplatin might therefore be due to, at least in part, higher intracellular platinum accumulation. For PNCs, a relationship between platinum accumulation and DNA platination was established, and increased DNA platination was correlated with increased toxicity [28].

High accumulation upon PN149 might be explained by the strong lipophilicity of the compound, as a correlation between the length of the axial ligand, which increases lipophilicity, and cellular accumulation has been observed for various PNCs [28]. It is discussed that, due to high lipophilicity, PNCs are mainly taken up by passive diffusion through the lipid bilayer, while active transport mechanisms by membrane transporters seem to play a minor role. As a consequence, the activity of specific transporters in the membranes of the proximal tubule cells would not affect the influx of PN149. For cisplatin, on the other hand, the involvement of membrane transporters in renal uptake and efflux has been shown. The organic cation transporter 2 (OCT2), which is part of the SLC family, is highly expressed in the basolateral membrane of PTECs and appears to be a major key player in the uptake of cisplatin in PTECs. A number of studies have shown a correlation between OCT2-mediated uptake and cisplatin-induced nephrotoxicity [42,53,54]. For oxaliplatin, uptake via OCT2 has also been observed, while carboplatin has no affinity to this transporter, which explains the lack of nephrotoxicity during carboplatin chemotherapy [49,55]. Oxaliplatin is taken up by OCT2 in kidney tubule cells, but also has no nephrotoxic potential. This might be explained by the high efflux of oxaliplatin out of tubule cells compared to the efflux of cisplatin. The SLC family members multidrug and toxin extrusion 1 and 2-K (MATE1/2-K) are implicated in the efflux of substances out of kidney cells [56]. Cisplatin shows no affinity for MATE2-K and has only low affinity for MATE1, whereas oxaliplatin represents a substrate for both MATE1 and MATE2-K, which will result in the efflux of oxaliplatin out of tubule cells and hence might explain the lack of nephrotoxicity [56]. Based on the different affinities of the platinum complexes towards the membrane transporters, one would expect differences in platinum levels following treatment with the three platinum drugs, which, however, was not the case (Table 1). In a comparable study, Yonezawa and colleagues analyzed the intracellular platinum accumulation following incubation with the three platinum-based compounds in HEK293 cells. Using 500 µM of each compound, they observed a comparable intracellular accumulation of cisplatin and oxaliplatin, while the intracellular accumulation of carboplatin was only a fraction of that [56]. In A498 kidney cancer cells, we also observed that at equimolar concentrations carboplatin showed substantially lower platinum accumulation than oxaliplatin and cisplatin, while equitoxic concentrations led to an accumulation similar to that of cisplatin [57]. However, in vivo animal studies revealed a considerably higher accumulation in rat kidneys following cisplatin treatment as compared to carboplatin or oxaliplatin [49]. Altogether, the data indicate that simple cell culture models obviously do not reflect the situation in the kidney adequately and therefore are not the best suited model to study the complex mechanisms of nephrotoxicity. Ludwig and colleagues investigated the influence of different cell culture models on the toxicity of cisplatin, carboplatin and oxaliplatin [58]. They cultivated the C7 clone of the canine kidney MDCK cell line in a Transwell^®^ system, which enables the differentiation of the basolateral and luminal side of epithelia, to investigate the influence of the side of application for platinum toxicity. Their results revealed that toxic effects of platinum complexes on renal epithelial cells depend on both the platinum complex and the side of application. Carboplatin and oxaliplatin were also toxic in kidney cells, as shown by the activation of caspase-3, but to a lesser extent than cisplatin. The strongest toxic effect was observed for cisplatin when it was applied on the basolateral side [58]. Altogether, the data again highlight the complexity of studying nephrotoxicity. Transport in renal proximal tubule cells is the most essential function in the kidney and therefore different membrane transporters are expressed in the basolateral and luminal membrane to guarantee the transcellular transport of endogenous compounds and xenobiotics. Hence, this function must be ensured in a cell culture study to investigate the nephrotoxic potential of a potential new drug. The gene expression of membrane transporters increases with the complexity of the cell culture model [59,60], and it is therefore recommended to test the nephrotoxic potential of a potential new drug in a more complex cell culture model. Nevertheless, to analyze the intracellular effects of platinum-based compounds in normal renal cells, the impact on cellular signalling pathways involved in the maintenance of genomic stability was analyzed in the cell culture model of ciPTEC.

### 3.4. Gene Expression Profiling

**Figure 3:**
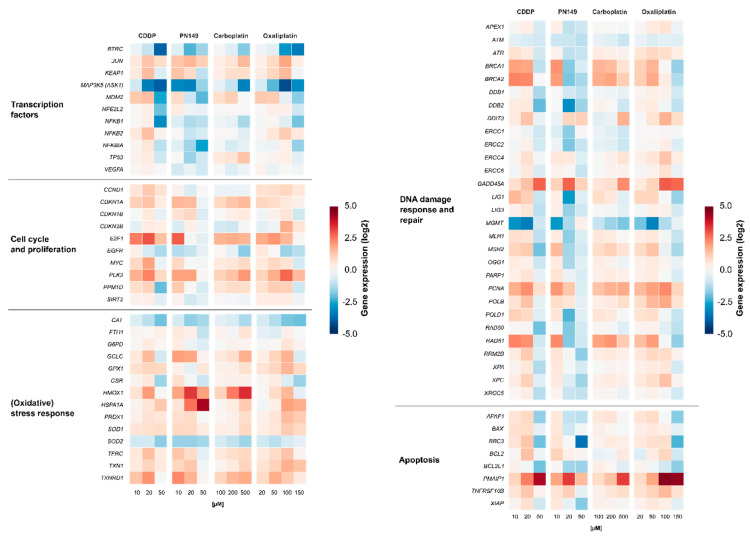
Gene expression profiling after treatment with platinum drugs. ciPTECs were treated with PN149 or cisplatin (10, 20, 50 µM), carboplatin (100, 200, 500 µM) or oxaliplatin (20, 50, 100 and 150 µM) for 24 h. Gene expression was determined by high-throughput RT-qPCR. Genes were grouped into the clusters of transcription factors, oxidative stress response, cell cycle and proliferation, DNA damage response/repair and apoptosis. Shown are the log2 mean values of three independent experiments normalized to the untreated control, whereby the control equals 0.

Gene expression profiles were generated to identify the cellular response of the experimental compound PN149 in comparison to the established chemotherapeutic drugs cisplatin, carboplatin and oxaliplatin in kidney proximal tubule cells. It is well understood that, due to its relatively high reactivity, cisplatin induces different cellular signaling pathways, which will ultimately lead to cell death [13]. Important signaling pathways that are implicated in cisplatin-induced nephrotoxicity are, among others, the MAPK signaling cascade as well as the p53 signaling cascade [61]. Detailed studies on the effect of platinum-based chemotherapeutics on renal cells and the involved signaling pathways, however, are rare. Comparative analysis of the expression of genes related to cellular transport, DNA repair and apoptosis was carried out in PTECs of the rat [48], while for human PTECs only data obtained for cisplatin are available [62]. We therefore extended these studies in ciPTECs. ciPTECs were treated for 24 h with different concentrations of the respective platinum compound, followed by quantitative high-throughput RT-qPCR to generate gene expression profiles of selected genes. The analyzed genes were clustered into groups of transcription factors, DNA damage response (DDR) and repair, cell cycle control, (oxidative) stress response and apoptosis. Relative gene expressions were calculated by normalizing the treated samples to the untreated control and are expressed as log2 values. The gene expression profiles are presented in a heatmap in Figure 3.

#### 3.4.1. Impact of Platinum Complexes on Genes Coding for Transcription Factors

Treatment with 20 µM PN149 led to widespread gene repression of genes related to transcription factors (Figure 3). 

Cisplatin at the highest concentration of 50 µM also resulted in a strong down-regulation of gene transcripts. Carboplatin, on the other hand, showed no general down-regulation, while for oxaliplatin widespread gene repression was restricted to the highest concentration used (150 µM). Widespread gene down-regulation might indicate pronounced cytotoxicity and hence impaired survival, which is supported by the findings regarding the cytotoxicity of the platinum compounds (Figure 2A) and was also confirmed by visual observation of the cells. Under viable conditions, all four platinum compounds led to a weak induction of *JUN* and *KEAP1*. *JUN* encodes the JUN protein subunit of the transcription factor AP-1, which is implicated in cell proliferation, cell differentiation and apoptosis [63]. Increased *JUN* expression therefore indicates an activation of AP-1 by the platinum complexes. *KEAP1*, as part of the KEAP1–Nrf2–ARE pathway of anti-oxidant gene regulation, might serve as an indicator for the induction of oxidative stress [64]. Positive modulations of the proliferation-associated *JUN* and *KEAP1* suggest the activation of cell cycle progression and induction of an oxidative stress response following exposure to PN149 and the chemotherapeutics in normal human kidney cells. However, a strong repression of *MAP3K5* coding for MAPKKK5/Ask1 kinase, which is part of the mitogen-activated protein kinase (MAPK) pathway, was also observed. MAPK pathways consist of cascades of serine/threonine kinases, which control cellular homeostasis, cell differentiation, cell proliferation and survival [65]. This observation would rather suggest an inhibitory effect on cell cycle progression by the platinum compounds. *NFKB2* was transcriptionally slightly enhanced by cisplatin and oxaliplatin, while repressed transcript levels were observed for PN149 and carboplatin. *NFKB2* encodes a precursor subunit of the transcription factor NFκB [66], which controls a number of cellular processes, among them angiogenesis, cell proliferation and inflammatory activity [67]. As inflammation is one of possible causes contributing to nephrotoxicity, the observations indicate a possible inflammatory response in kidney proximal tubule epithelia by cisplatin and oxaliplatin, but a lack of it following treatment with carboplatin and PN149.

#### 3.4.2. Impact of Platinum Complexes on Genes Related to Oxidative Stress Response and Inflammation

**Figure 4:**
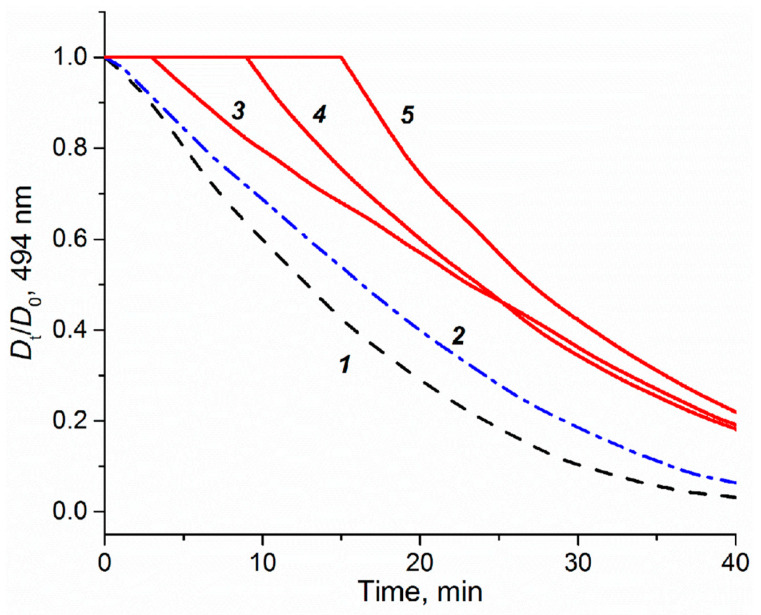
Effect of platinum complexes on the consumption of fluorescein (10 µM) induced by peroxyl radicals generated from AAPH (50 mM) in phosphate buffer (50 mM, pH 7.4) at 37 °C. The consumption of fluorescein was followed by visible absorption at 494 nm in the absence (*1*) and presence of cisplatin (*2*, 10 µM) and PN149 (*3*, 10 µM), (*4*, 25 µM), and (*5*, 50 µM).

The impact of the platinum compounds on the expression of oxidative stress response genes is summarized in Figure 3. The ROS-inducible *HMOX1* gene was strongly induced by PN149 and carboplatin, while slightly lesser effects were observed in the case of cisplatin and oxaliplatin. The HMOX1 protein has a protective effect against oxidants, hence the lack of HMOX1 in *HMOX1* KO mice resulted in increased cisplatin-induced nephrotoxicity [68]. PN149 led to a strong concentration-dependent increase in the heat-shock sensitive *HSPA1A*, which was, however, restricted to cytotoxic conditions, whereas a less pronounced effect was observed for the other three platinum compounds. The transcription of *HMOX1* and *HSPA1A* is mediated via different redox-sensitive transcription factors such as Nrf2 and NFκB, which makes *HMOX1* and *HSPA1A* marker genes for ROS generation. Therefore, gene expression analysis indicates ROS production by PN149 and, to a lesser extent, by the three chemotherapeutic platinum drugs in normal proximal tubule epithelia of humans. With regard to genes associated with the anti-oxidant defense system, a significant up-regulation was observed for *GCLC* and *TXNRD1*, which code for anti-oxidative enzymes, while weak gene modulation was observed for *SOD1, TXN1, PRDX1, TFRC,* and *GPX,* which are all related to the glutathione and thioredoxin system. On the other hand, strong down-regulation was observed for *CAT* and *SOD2* in the case of all four compounds, suggesting the depletion of anti-oxidant enzymes. Experiments in rodents showing significantly reduced renal activity of superoxide dismutase and catalase following cisplatin treatment support the gene expression analysis on the functional level [69]. On the other hand, Wilmes and co-authors observed an increased expression of *CAT* in RPTEC kidney cells, while *SOD2* transcript levels were also reduced [62], which is in line with observations of reduced amounts of *SOD2*-encoded MNSOD protein in cisplatin-induced in vivo nephrotoxicity [70]. Altogether, the gene expression analysis revealed the induction of genes associated with the anti-oxidant defense at the transcriptional level for all compounds, suggesting an oxidative stress response in normal proximal tubule cells due to ROS formation. ROS are discussed as one factor contributing to cisplatin-induced nephrotoxicity [71]. ROS formation following cisplatin treatment was observed only in epithelial cells of the S3 segment of the proximal tubule, which is the main place of cisplatin-induced nephrotoxicity, but not in cells of the medullary collecting tube, which are not a target for cisplatin nephrotoxicity [72]. Furthermore, it has been demonstrated that anti-oxidants reduce cisplatin-induced nephrotoxicity in animal models [73]. An anti-oxidative potential is also attributed to nitroxyl radicals, which mimic the activity of superoxide dismutase and/or stoichiometrically react with ROS [74,75]. PNCs were therefore designed by the addition of an aminonitroxyl group to platinum, with the aim to develop a platinum compound with a potential anti-oxidant property and hence reduced dose-limiting nephrotoxicity. However, based on the toxicity data of the present study, it is difficult to conclude on an anti-oxidative and hence protective potential by PN149. Experiments were therefore carried out to investigate for a possible anti-oxidant capacity of PN149 in comparison to cisplatin. The method to assess anti-oxidant capacity is based on the ability of anti-oxidants to scavenge peroxyl radicals, RO_2_^•^, generated by the azo-initiator AAPH and thus to inhibit the consumption of fluorescein used as a probe. Fluorescein consumption is easily monitored spectrophotometrically at 494 nm [35]. PN149 effectively inhibited the consumption of fluorescein and produced a clear lag phase directly proportional to the concentration of the complex (Figure 4, curves 3–5). Cisplatin was also not inert in this investigation, but only slightly slowed down the consumption of fluorescein (curve 2). This weak effect of cisplatin probably explains the behavior of curves 3–5 after ~20 min of reaction. By this time, the nitroxyl radical of PN149 is consumed and the course of curves 3–5 differs only insignificantly. Interestingly, the kinetics of fluorescein consumption in the presence of the Pt^IV^ complex JM216 (10 M) practically coincided with curve 2 for cisplatin (data not shown), which might indicate that the Pt^II^/Pt^IV^ metals do not participate in the reaction and some part of RO_2_^•^ radicals react with the amino groups of the platinum complexes. Altogether, PN149 showed an oxygen radical absorbance capacity in a simple test model. However, this does not necessarily indicate such an activity in a complex system such as the cell, as gene expression analysis showed the modulation of genes related to ROS production and oxidative stress response by PN149, and PN149 was highly cytotoxic in ciPTECs.

**Figure 5:**
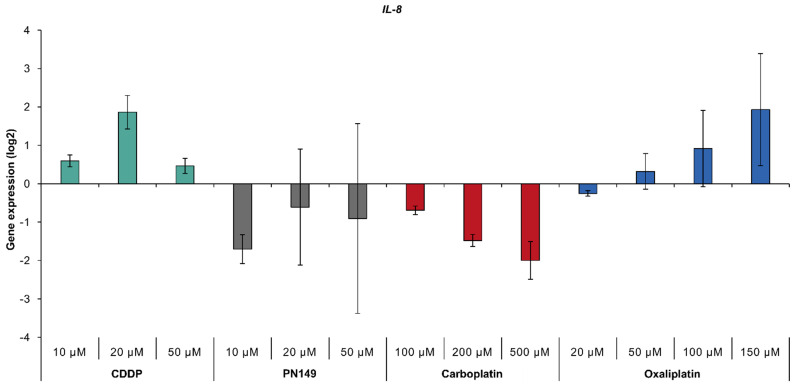
Gene expression of *IL-8* gene. ciPTECs were treated with PN149 or cisplatin (10, 20, 50 µM), carboplatin (100, 200, 500 µM) or oxaliplatin (20, 50, 100 and 150 µM) for 24 h. Gene expression was determined by high-throughput RT-qPCR. Shown are the log2 mean values of three independent experiments normalized to the untreated control, whereby the control equals 0.

Inflammatory processes, mediated by TNFα and NFκB, have also been implicated in the toxicity of cisplatin to kidney cells [4,12,13,14,15,16]. Possible inflammatory activity by the platinum compounds was investigated via the expression of *IL-8*, which serves as a marker for inflammation. The treatment of ciPTECs with cisplatin and oxaliplatin increased transcript levels of *IL-8*, while PN149 and carboplatin led to repressed gene expression (Figure 5), suggesting a pro-inflammatory response in human renal proximal tubule epithelia by cisplatin and oxaliplatin, but not by PN149 and carboplatin. As inflammatory processes have been linked to ROS-induced oxidative stress [76], the lack of an inflammatory response by PN149 might be an indication of the oxygen radical absorbance capacity of PN149 in a cellular system. In addition, the expression of inflammatory genes such as *IL-1*, *IL-6* and *IL-8* is controlled, among other factors, by NFκB [67], which is in line with the increased expression of the NFκB subunit gene *NFKB2* upon treatment with cisplatin and oxaliplatin (Figure 3).

#### 3.4.3. Impact of Platinum Complexes on Genes Related to Cell Cycle Control 

PN149 and the three chemotherapeutics affected the expression of genes associated with cell cycle regulation and proliferation (Figure 3). The induction of gene expression was most pronounced for *E2F1*, *PLK3* and *CDKN1A* after treatment with the respective compounds, while *CCND1*, *MYC* and *PPM1D* were enhanced to a lesser amount. Under physiological conditions, only 1% of kidney tubular epithelial cells are in a state of proliferation, while most of the cells are predominantly resting in the G_0_-phase of the cell cycle [77]. However, upon genotoxic stress and DNA damage, the DDR system in kidney cells is activated, inducing entry into the cell cycle [78]. *E2F1*, *CCND1* and MYC are implicated in the G_1_-to-S transition [79,80,81]; therefore, gene expression analysis indicates progression from the semi-permanent G_0_-phase into the cell cycle in platinum-damaged ciPTECs. However, increased expression of *CDKN1A* coding for the cyclin-dependent kinase inhibitor p21 would rather suggest cell cycle arrest following platinum treatment. The activation of cell cycle arrest and DNA repair will enable the cells to remove damage from the DNA to make sure that the fidelity of the DNA is restored before entry into the S- and M-phase. p21 is implicated in both arrest at the G_1_-to-S transition and inhibition of the G_2_-to-M transition [82]. In tubular epithelial cells, p21-mediated blockade of the G_2_-to-M transition in response to damage has been associated with nephrotoxicity and chronic kidney disease [83].

Taken together, at viable concentrations PN149 and the chemotherapeutic platinum drugs might lead to an entry into the cell cycle followed by the activation of cell cycle checkpoints to ensure that DNA damage is repaired and DNA fidelity is intact before further progressing through the cell cycle [84]. These results confirm previous studies reporting the activation of cell cycle regulation by cisplatin in the rodent NRH-52E kidney cell line and in vivo animal models of rats, demonstrated by enhanced expression of proliferation-related p21 on the gene and protein level, which was both dependent and independent of p53 [62,85,86].

#### 3.4.4. Impact of Platinum Complexes on Genes Related to DNA Damage Response/Repair and Apoptosis

With regard to genes related to DNA damage response and repair, treatment with non-toxic concentrations of the platinum compounds led to enhanced expression of *GADD45A* and *PCNA*. *GADD45A* and *PCNA* are target genes of p53; therefore, enhanced transcript levels indicate a p53-dependent DNA damage response following platinum treatment in normal kidney tubule epithelial cells. Non-toxic concentrations of the platinum compounds also led to increased expression of *BRCA1*/*BRCA2* and *RAD51*, which are part of homologous recombination double strand break (DSB) repair. It is discussed that DSBs might arise during the processing of DNA interstrand crosslinks (ICLs), which are induced by common agents such as mechlorethamine and also cisplatin. However, no DSBs were detected following cisplatin treatment [87]. On the other hand, γH2AX staining, which serves as a marker for DSBs, was observed in various cancer cell lines [88]. One could envisage that the processing of cisplatin-induced ICLs leads to the formation of ICL repair-associated DSB-intermediates, which are targets of homologous recombinational DSB repair in normal kidney tubule cells. BRCA1/BRCA2 and RAD51 proteins have been implicated in the repair of ICLs [89], which is in line with observed increased transcript levels of *BRCA1*/*BRCA2* and *RAD51* following treatment with PN149 and the chemotherapeutic drugs, indicating ICL repair in normal kidney epithelial cells. Furthermore, the transcription of *MSH2, POLB* and *POLD1*, which are implicated in mismatch repair, base excision repair and nucleotide excision repair [90,91,92], respectively, was increased by PN149 and the chemotherapeutic drugs in ciPTECs, indicating the activation of the DDR system in normal epithelial kidney cells treated with platinum compounds under viable conditions. Using cytotoxic concentrations of PN149, cisplatin and oxaliplatin, widespread gene repression was observed, while carboplatin did not lead to a general repression of transcript levels (Figure 3).

In vivo studies suggested that cisplatin leads to nephrotoxicity via the induction of apoptosis and, to a lesser extent, necrosis in proximal tubule epithelia [93]. Cisplatin-induced apoptosis may be triggered through the extrinsic death receptor pathway or the intrinsic mitochondrial pathway [94]. ciPTECs treated with the respective platinum compounds showed modulation of pro- and anti-apoptotic genes (Figure 3). The gene expression level of the anti-apoptotic gene *BCL2* was slightly induced, which would indicate the prevention of apoptosis upon platinum drug treatment in proximal tubule cells. On the other hand, genes affecting both the extrinsic and the intrinsic apoptotic pathway were also induced by the platinum drugs. A strong concentration-dependent increase in the gene expression of the pro-apoptotic gene *PMAIP1*, encoding the protein Noxa, was observed following treatment with cisplatin, carboplatin and oxaliplatin. PN149-induced *PMAIP1* levels, on the other hand, peaked at 20 µM, followed by a decrease at 50 µM. Under these conditions, strong cytotoxicity was observed 48 h post-treatment (Figure 2A). Platinum treatment also increased transcript levels of the pro-apoptotic genes *BAX*, *BBC3* and *TNFRSF10B*, however, to a lesser extent and restricted to viable concentrations. With respect to pro-apoptotic signaling, effects were observed for both the intrinsic pathway (*PMAIP1, BAX, BBC3)* and extrinsic pathway (*TNFRSF10B*), suggesting that PN149 and the chemotherapeutic drugs induce apoptosis in proximal tubule cells, which is executed via the intrinsic and extrinsic pathway. Furthermore, *BAX*, *PMAIP1* and *TNFRSF10B* are transcriptionally regulated by p53 [95], which suggests a critical role for p53 in platinum-induced apoptosis in proximal tubule cells. With regard to cisplatin, carboplatin and oxaliplatin, the induction of apoptosis in kidney cells has been confirmed on the functional level. Jiang and co-authors observed the activation of p53, followed by strong expression of *BBC3*-encoded PUMA and activation of BAX, leading to apoptosis in RPTC normal rat kidney proximal tubular cells [96]. Likewise, Krüger and co-authors observed an increased expression of the apoptotic gene *BAX* following treatment with cisplatin, carboplatin and oxaliplatin in rat NRK-52E kidney cells, which was accompanied by the activation of caspases 3 and 7 on the functional level [48].

Taken together, our data indicate an induction of the DDR system at the transcriptional level by PN149 and the established platinum chemotherapeutics in normal human tubule kidney cells. At viable concentrations, repair pathways are activated for cells to cope with the damage. Severe damage, however, will trigger apoptotic cell death via the extrinsic and intrinsic apoptotic cascade and eliminate the injured tubular epithelial cells.

### 3.5. Comparison of Gene Expression Profiles of Cisplatin in ciPTECs vs. Normal Renal Tissue Cells 

The observations above illustrate the problems one encounters when performing in vitro nephrotoxicity studies. ciPTECs, which retained characteristics of proximal tubule epithelia, were used as a permanent cell line for the current investigations. To validate the findings obtained with ciPTECs in a model that resembles the in vivo situation more closely, primary cells derived from human kidneys were used. The treatment of primary kidney cells with 20 µM cisplatin for 24 h resulted in platinum levels of 25 ng Pt/10^6^ cells as compared to 17 ng Pt/10^6^ cells, which were observed in ciPTECs (Table 2). Possibly, differences in expression levels of membrane transporters can account for the higher platinum levels in primary kidney tubule cells, as permanent cell lines often show reduced or missing expression of membrane transporters.

**Figure 6:**
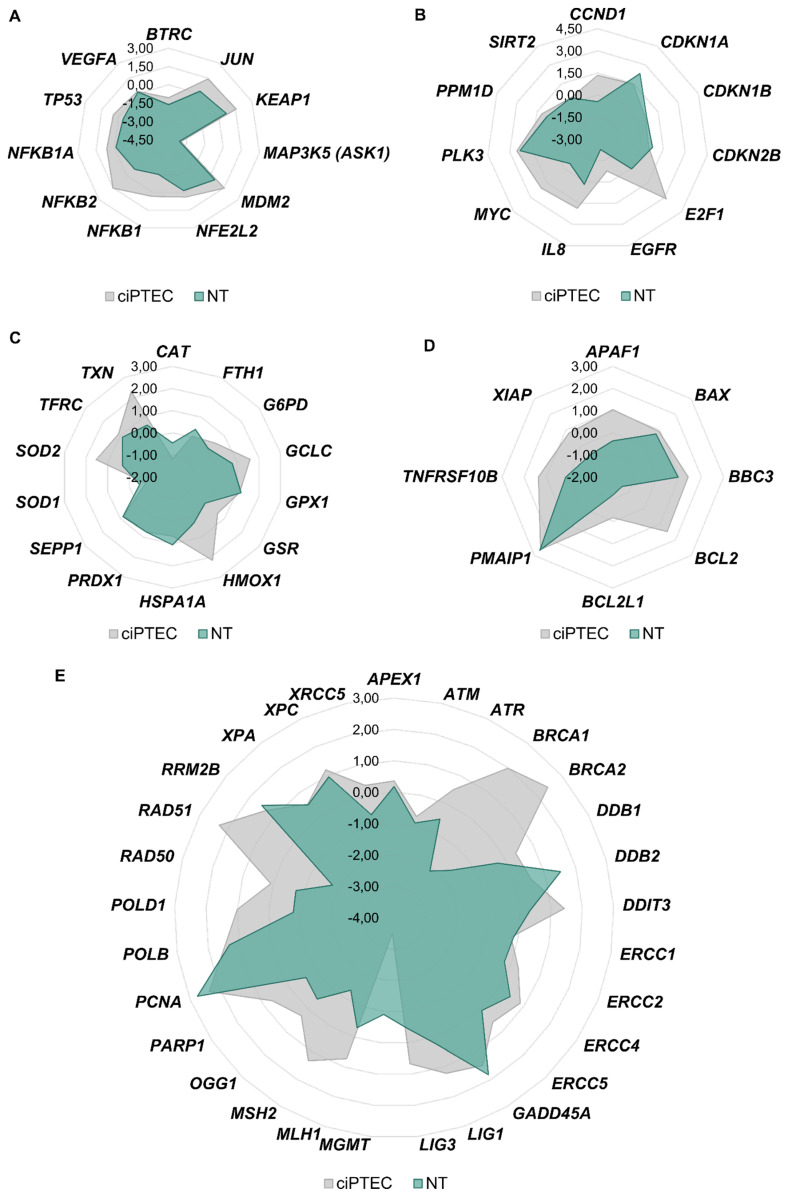
ciPTECs and normal kidney cells were treated with 20 µM cisplatin for 24 h, and gene expression analysis was performed with high-throughput RT-qPCR. Genes were grouped into the clusters of transcription factors (**A**), cell cycle and proliferation (**B**), oxidative stress response (**C**), apoptosis (**D**) and DNA damage response/repair (**E**). Gene clusters were arranged in network diagrams.

Gene expression analysis was performed to establish expression profiles associated with cisplatin nephrotoxicity, and gene clusters were arranged in network diagrams to compare the data of the primary kidney cells with ciPTECs (Figure 6).

With regard to genes related to transcription factors, DNA damage response, cell cycle control, (oxidative) stress response and apoptosis, a comparable impact on gene expression was observed in both cellular models. Overall, expression levels were more pronounced in ciPTECs, suggesting a more sensitive reaction of the permanent cell line to cisplatin. With regard to genes related to DNA repair, however, a profound difference between the two model systems was observed. While increased expression of several repair-related genes was observed in cisplatin-treated ciPTECs, reduced transcript levels were seen in normal tissue (Figure 6). This might be due to the degree of differentiation of the cells. The down-regulation of repair genes and hence attenuated global genome DNA repair is observed in terminally differentiated cells, which is explained by the reduced necessity to remove DNA lesions from the non-essential mass of the DNA [97]. In line with the gene expression analysis of DNA repair genes, a reduced repair capacity has been observed in kidney tissue affected by AKI, which often occurs as a result of cisplatin chemotherapeutic treatment [98].

In general, primary renal tissue is best suited for investigation regarding the kidney, as it offers the closest reflection of the in vivo situation. However, a number of limitations must be taken into account. Inter-individual variations of tissue donors need to be considered for the interpretation of experimental results. Furthermore, cells derived directly from the human kidney are limited. Therefore, the permanent cell line ciPTEC offers a good alternative for experimental studies regarding the impact of compounds on cellular processes in kidney cells. However, simple in vitro cell culture models are not best suited to investigate the complex mechanisms of nephrotoxicity. In the present study, all four platinum compounds were toxic in ciPTECs and evoked a similar cellular response. This, however, does not reflect the clinical situation, where carboplatin and oxaliplatin show little or no toxicity in proximal tubule epithelia in cancer patients. Caution should therefore be used to extrapolate the in vitro toxicity data of the experimental compound PN149 to an in vivo situation, and definite conclusions about the nephrotoxic potential of PN149 should not be drawn on the basis of the present study. As the kidney is a highly complex organ, whose function relies on transport processes, a more complex model, such as Kidney-on-a-chip, which enables more realistic transport processes in kidney cells, might offer a better suited system for complex nephrotoxicity studies [99]. Alternatively, tubule suspensions or proximal tubular-like cells derived from human-induced pluripotent stem cells might be used as a proximal tubular cell model [100]. Nonetheless, the information gained in the current study about the impact of the platinum drugs on pathways related to genomic stability might help for the design of new platinum drugs that are safer for patients. 

## 4. Conclusions

Cisplatin is one of the major drugs used for cancer treatment. Unfortunately, its use is restricted by toxic side effects, especially dose-limiting nephrotoxicity. Therefore, new platinum analogues are designed, aimed at reducing or removing nephrotoxicity. The mode of action of the experimental platinum compound PN149 was elucidated on the transcriptional level in normal human kidney tubule epithelial cells in comparison to the clinically approved platinum chemotherapeutics cisplatin, carboplatin and oxaliplatin. The expression of genes related to genomic stability revealed an impact of PN149 on the oxidative stress response, DNA damage response/repair, cell cycle regulation and apoptosis, similar to that observed for the chemotherapeutic drugs. However, no pro-inflammatory response, which was shown to be of major importance for cisplatin-induced nephrotoxicity, was observed in PN149-treated ciPTECs, which might indicate the reduced nephrotoxic potential of PN149. PN149 was highly cytotoxic to the human proximal tubule ciPTEC cell line, accompanied by high levels of intracellular platinum accumulation. Intracellular platinum levels were similar for cisplatin, carboplatin and oxaliplatin, which does not reflect clinical observations in cancer-treated patients. The present study highlights the problems of performing nephrotoxicity studies in vitro and stresses that caution has to be taken when extrapolating data from an in vitro model system to the in vivo situation.

## Figures and Tables

**Table 1:** Calculated IC_50_ values and platinum accumulation following incubation of ciPTECs with 50 µM of either cisplatin, PN149 or oxaliplatin or 300 µM carboplatin for 2 h. Shown are mean values from at least three independent determinations ± standard deviation.

Platinum Compound	IC_50_ [µM]	ng Pt/10^6^ Cells
Cisplatin	13	17 ± 5
PN149 Oxaliplatin Carboplatin	6 51 175	244 ± 49 11 ± 2 18 ± 2

**Table 2:** Platinum accumulation in ciPTECs and primary kidney cells following incubation with 50 µM cisplatin for 2 h. Shown are mean values from at least three independent determinations ± standard deviation.

Cellular Model	ng Pt/10^6^ Cells
ciPTEC	17 ± 5
Primary kidney cells	25 ± 5

## Abbreviations

AAPH	2,2′-azobis(2-amidinopropane) dihydrochloride
AAS	atomic absorption spectroscopy
AKI	acute kidney injury
DDR	DNA damage response
DSB	double strand break
ICL	interstrand crosslink
PB	phosphate buffer
PNC	platinum(IV)–nitroxyl complex
PN149	e-ammine-d-(3-amino-2,2,5,5-tetramethylpyrrolidine-1-oxyl)-a,f-bis(butanoato)-b,c-dichloroplatinum(IV)
PTECs	proximal tubule epithelial cells
RCC	relative cell count
ROS	reactive oxygen species
SLC	solute carrier
